# Exosomes from bone marrow mesenchymal stem cells ameliorate glucocorticoid-induced osteonecrosis of femoral head by transferring microRNA-210 into bone microvascular endothelial cells

**DOI:** 10.1186/s13018-023-04440-x

**Published:** 2023-12-07

**Authors:** Che Zheng, Yuangang Wu, Jiawen Xu, Yuan Liu, Jun Ma

**Affiliations:** 1 Department of Orthopedic Surgery and Orthopedic Research Institute, West China Hospital, Sichuan University, 37# Guoxue Road, Chengdu, 610041 People’s Republic of China; 2 Department of Orthopedic Surgery, Chengdu Second People’s Hospital, Chengdu, Sichuan Province People’s Republic of China

## Abstract

**Objectives:**

Bone microvascular endothelial cells (BMECs) played an important role in the pathogenesis of glucocorticoid-induced osteonecrosis of femoral head (GCS-ONFH), and exosomes derived from bone marrow mesenchymal stem cells (BMSC-Exos) may provide an effective treatment. This study aimed to evaluate the effects of BMSC-Exos and internal microRNA-210-3p (miRNA-210) on GCS-ONFH in an in vitro hydrocortisone-induced BMECs injury model and an in vivo rat GCS-ONFH model.

**Methods:**

BMECs, BMSCs and BMSC-Exos were isolated and validated. BMECs after the treatment of hydrocortisone were cocultured with different concentrations of BMSC-Exos, then proliferation, migration, apoptosis and angiogenesis of BMECs were evaluated by CCK-8, Annexin V-FITC/PI, cell scratch and tube formation assays. BMSCs were transfected with miRNA-210 mimics and miRNA-210 inhibitors, then BMSC-Exos^miRNA−210 mimic^ and BMSC-Exos^miRNA−210 inhibitor^ secreted from such cells were collected. The differences between BMSC-Exos, BMSC-Exos^miRNA−210 mimic^ and BMSC-Exos^miRNA−210 inhibitor^ in protecting BMECs against GCS treatment were analyzed by methods mentioned above. Intramuscular injections of methylprednisolone were performed on Sprague–Dawley rats to establish an animal model of GCS-ONFH, then tail intravenous injections of BMSC-Exos, BMSC-Exos^miRNA−210 mimic^ or BMSC-Exos^miRNA−210 inhibitor^ were conducted after methylprednisolone injection. Histological and immunofluorescence staining and micro-CT were performed to evaluate the effects of BMSC-Exos and internal miRNA-210 on the in vivo GCS-ONFH model.

**Results:**

Different concentrations of BMSC-Exos, especially high concentration of BMSC-Exos, could enhance the proliferation, migration and angiogenesis ability and reduce the apoptosis rates of BMECs treated with GCS. Compared with BMSC-Exos, BMSC-Exos^miRNA−210 mimic^ could further enhance the proliferation, migration and angiogenesis ability and reduce the apoptosis rates of BMECs, while BMECs in the GCS + BMSC-Exos^miRNA−210 inhibitor^ group showed reduced proliferation, migration and angiogenesis ability and higher apoptosis rates. In the rat GCS-ONFH model, BMSC-Exos, especially BMSC-Exos^miRNA−210 mimic^, could increase microvascular density and enhance bone remodeling of femoral heads.

**Conclusions:**

BMSC-Exos containing miRNA-210 could serve as potential therapeutics for protecting BMECs and ameliorating the progression of GCS-ONFH.

**Supplementary Information:**

The online version contains supplementary material available at 10.1186/s13018-023-04440-x.

## Introduction

Osteonecrosis of femoral head (ONFH) was one of the most common chronic musculoskeletal disorders and a leading cause of disability globally [1, 2], and long-term application of high-dose glucocorticoid (GCS) was the most common causes for non-traumatic ONFH. Recently, many advances have been made in joint-preserving treatments such as osteotomies, core decompression and non-vascularized or vascularized bone grafting, but a variety of poor prognostic factors might result in treatment failure and conversion to total hip arthroplasty (THA) [3–5]. Although THA can improve the quality of life of patients with GCS-ONFH [6, 7], it only represented an end-stage treatment surgery with risk of reoperations. In addition, the majority of patients suffering from GCS-ONFH are young and in constant need of multiple surgeries over their entire life‐time [8]. Thus, it is important to elucidate the pathophysiology of glucocorticoid‐induced ONFH and find new ways of preventing its occurrence.

The etiology of GCS-ONFH is complex, with various factors including fat cell enlargement, rise in intraosseous pressure and osteoporosis which might take part in the pathogenesis of GCS-ONFH [9, 10]. However, risk factors are related to ischemia of femoral head, as corticosteroids overuse can result in fat emboli, intravascular coagulation and thrombotic occlusion [11, 12]. In addition, the previous studies suggested that glucocorticoids could directly harm the bone microvascular endothelial cells (BMECs) in the femoral heads, causing vasoconstriction, thrombus formation and disturbance of the coagulation–fibrinolysis system [13, 14]. BMECs were arranged in the inner layer of blood vessels and played a crucial role in vascular homeostasis and angiogenesis [15]. BMECs could secrete a variety of angiogenesis promoters and markers, including vascular endothelial growth factor (VEGF), CD31, von Willebrand factor (vWF) and platelet-derived growth factor B (PDGF-B) [16, 17]. Therefore, maintaining the blood circulation within the femoral head and inhibiting BMECs injury were a key step to preventing glucocorticoid‐induced ONFH.

Bone marrow mesenchymal stem cells (BMSCs) play an important role in promoting angiogenesis and regeneration due to their multidirectional differentiation potential and self-renewal ability [18]. Although BMSCs have a wide range of applications and sufficient supply, BMSC-related complications have attracted widespread attention. BMSCs can differentiate into osteoblast, and locally implantation of BMSCs might result in ectopic ossification [19]. In addition, clinical studies showed that intravenous injection of BMSCs had potential risk of distal vascular embolism and arrhythmia. In vivo studies also showed that application of BMSCs can cause cell embolism in organs such as brain, heart, lung, liver, kidney and spleen in rats [20–22]. Recent studies showed that the therapeutic effect of stem cell transplantation was mainly due to their secretory behavior [23, 24]. Among these secreted biochemicals, exosomes seemed to play an important role in tissue repair and were involved in transportation of functional biochemicals, such as cytokines, RNAs and proteins [23, 25]. Transplantation of exosomes has been confirmed to exert similar therapeutic effects to direct stem cell transplantation in tissue repair [26, 27]. Moreover, such an alternative strategy avoids all the issues caused by direct stem cell transplantation. Transplantation of exosomes secreted by stem cells might, therefore, be a promising treatment option for GCS-ONFH.

The previous studies showed that microRNAs (miRNAs) account for approximately 13% of the total RNA in BMSC-Exos [28, 29], and miRNAs are related to the pathogenesis of tendon injuries and osteoarthritis [30–32]. Single-stranded nucleic acids can inhibit the expression of target genes by binding to the untranslated region of target miRNA molecules [33, 34]. A variety of miRNAs inside BMSC-Exos have been proved to be effective in protecting against ischemia diseases [35, 36]. Similarly, BMSC-Exos contained miRNA-210 and miRNA-210 have been proved to be effective in promoting the proliferation of endothelial cells, angiogenesis and reducing apoptosis [37]. Besnier et al. found that miRNA-210 could enhance the therapeutic potential of bone marrow-derived circulating proangiogenic cells to limb ischemia [38]. Zhang et al. found that miRNA-210 in exosome could serve as an angiogenic therapy for cerebral ischemia in mice [39]. In addition, miRNA-210 might take part in the pathogenesis of GCS-ONFH. miRNA microarray with ONFH tissues and the adjacent normal tissues showed that miRNA-210 was differentially expressed [40], and miRNA-210 was present in cells surrounding osteonecrosis [41].

Considering the important role of BMECs in the pathogenesis of GCS-ONFH and therapeutic potential of miRNA-210 in BMSC-Exos to this disease, we hypothesize that BMSC-Exos could ameliorate the progression of GCS-ONFH, and miRNA-210 could enhance the effects of angiogenesis. In vitro and in vivo experiments were used to test this hypothesis.

## Materials and methods

### Isolation and culture of BMSCs

Three-week-old male Sprague–Dawley rats were killed, and then, the femur and tibia were removed aseptically. The ends of the femur and tibia were cut with sterile scissors, and the bone marrow cavity was flushed from the bone shaft with Dulbecco’s modified Eagle medium (DMEM; Gibco) containing 10% fetal bovine serum (FBS; Gibco). The bone marrow cavity flush fluid was collected and centrifuged for 5 min at 1200 rpm. The cells at the junction were collected and cultured in DMEM medium (Gibco) containing 10% FBS (Gibco) at 37 °C in a humidified atmosphere with 5% CO_2_. The culture medium was changed every 48 h until passaging. Expression levels of cell surface markers were analyzed to confirm the characteristics of BMSCs as previously described [42, 43].

### Isolation and culture of BMECs

Eight-week-old female Sprague–Dawley rats were sacrificed, then the femoral head was dissected and cut into 1-mm^3^ pieces. The bone debris was transferred into a 10-ml centrifuge tube and washed three times by DMEM medium for 5 min to remove the fat tissue and blood cells. After removing the supernatant, the bone debris was moved to another 10-ml centrifuge tube containing and digested by 5-ml 0.2% collagenase I (4-ml DMEM + 1 ml 1% collagenase I) for 30 min on an orbital shaker at 37 °C. Then, 3-ml 0.25% trypsin were added for continued digestion for 5 min. The enzyme solution was then inactivated by adding DMEM containing 10% FBS. A 70-um cell filter was used for removing undigested tissue remnants. After centrifuged at 430 g for 6 min, the supernatant was discarded, and BMECs were resuspended and maintained in endothelial cell medium (ECM; Sciencell) containing 15% FBS and 1% endothelial cell growth supplement (ECGS) at 37 °C in a humidified atmosphere with 5% CO2. The culture medium was changed every 48 h until passaging. Expression levels of the markers CD31, CD133 and vWF [44, 45] were confirmed by immunofluorescence.

### Isolation of exosomes from BMSCs

BMSC-Exos were isolated by using an ultracentrifugation approach according to the previously described protocol [46, 47]. In short, when BMSCs at P3 reached 80% confluence, the DMEM medium was replaced with a medium containing 15% Exo-free FBS (Gibco), and the cells were cultured for 48 h to produce Exo-rich supernatant. Then, the supernatant was centrifuged at 2000*g* for 15 min at 4 °C to remove dead cells and then transferred to a fresh centrifuge tube and centrifuged at 10,000*g* (Avanti JXN-26; Beckman) for 30 min at 4 °C to remove cellular debris. After passing through a 0.22-mm filter, the supernatant was moved into another ultracentrifuge tube and centrifuged at 100,000*g* (Optima XPN-100; Beckman) for 120 min at 4 °C. Isolated BMSC-Exos were resuspended in cold phosphate-buffered saline (PBS) and stored at − 80 °C. A bicinchoninic acid (BCA) kit (Beyotime) was used to evaluate the concentration of BMSC-Exos. Transmission electron microscopy (TEM), nanoparticle tracking analysis (NTA) and Western blotting were used to analyze the morphology, diameter and surface markers of BMSC-Exos.

### Establishment of the GCS-induced BMECs injury model and treatment

Before the experiment, 1 × 10^5^ BMECs were seeded into 6-well plates, which contained 2-mL complete medium. BMECs injury model was induced in normal BMECs at 70%–80% confluence by culturing in serum-free medium with 0.1-mg/mL hydrocortisone (Beyotime) for 24 h [44, 45, 48]. Then, the medium was replaced with serum-free medium containing 10-ug/ml, 50-ug/ml or 100-ug/ml BMSC-Exos incubated before the harvest and further testing. Finally, BMECs after the treatment of hydrocortisone were incubated with serum-free medium containing BMSC-Exos, BMSC-Exos^miRNA−210 mimic^ or BMSC-Exos^miRNA−210 inhibitor^ for further testing.

### Cell proliferation assay

The proliferation of BMECs stimulated by exosomes was measured by using a Cell Counting Kit-8 (CCK-8; Elabscience). Briefly, after stimulation with hydrocortisone and treatment with exosomes, 5000 cells/well were incubated in 96-well dishes for 24 h. Then, 10-mL CCK-8 was added to each well and incubated for 2 h. Cell proliferation was evaluated by absorbance values at 450 nm with a spectrophotometer (Bio-Tek Instruments).

### Cell scratch assay

About 5 × 10^5^ BMECs per well were incubated in 6-well plates till they grew to the required confluence, then they were treated with different exosomes. A 200‐μL pipette tip was used to create a scratch on a cell monolayer. At 0, 24 and 48 h, the width of the scratch was measured, and the percentage of scratch recovery was determined.

### Cell apoptosis assay

An Annexin V-FITC/PI (fluorescein isothiocyanate, FITC; propidium iodide, PI) kit (Elabscience) was used to assess the rate of BMECs apoptosis based on the manufacturer’s protocol. In short, BMECs were centrifuged for 5 min at 1000 rpm, washed twice using cold PBS and resuspended in 500-mL binding buffer containing 10-mL PI (20 mg/mL) and 5-mL Annexin V-FITC. After incubating for 5 min in the darkness, the cells were assessed using a flow cytometer (LSRFortessa; Becton Dickinson) to quantify the rate apoptosis.

### Tube formation assay

A tube formation assay was performed to investigate the BMECs network formation. After different treatments, BMECs were seeded onto Matrigel-coated 24-well plates at a density of 1 × 10^5^ cells per well and cultured for 24 h. Then, the tube formation was visualized under a microscope (Nikon Corporation), and the number of branches of the associated tubes was calculated and compared among various groups.

### Western blotting

Radioimmunoprecipitation assay (RIPA) lysis buffer (Beyotime) was used to extract the total proteins from cocultured cells, and the total protein concentration in each extract was detected using a BCA protein assay kit (Beyotime). The extracted proteins were then mixed with a loading buffer (Pierce) and boiled at 100 °C for 10 min. Equal amounts of protein were separated by 12.5% sodium dodecyl sulfate polyacrylamide gel electrophoresis and transferred to a polyvinylidene difluoride membrane (Hybond). After blocking in 5% skim milk for 1 h at room temperature, the membrane was probed with primary antibodies at 4 °C overnight. The membrane was then incubated with secondary antibodies (ABclonal; 1:5000) at 37 °C for 1 h. Chemiluminescent signals were generated using an enhanced chemiluminescence (ECL) imaging kit (Thermo Fisher).

### Real-time polymerase chain reaction

Total RNA was isolated from treated BMSCs and BMECs using TRIzol reagent (Invitrogen), and then, 1-μg RNA was reverse‐transcribed into cDNA using a TaqMan Reverse Transcription Kit (Applied Biosystems). Next, 2 μl of cDNA was used as a template for the qRT‐PCR assay, which was performed using SYBRs GREEN PCR Master Mix (Thermo Fisher) according to the manufacturer's instructions. MicroRNA-210 relative expression to U6 was determined using the 2‐ΔΔCt method [49], and the primers used are shown in Additional file 1: Table S1.

### BMSC-Exos uptake assay

BMECs were cultured in 6-well plates with 2.5 × 10^6^ cells per well. About 4-ul PKH26 (Sigma) was added to 1-ml exosomes suspension, after 5-min incubation, 1% bovine serum albumin (BSA) was used to stop the reaction system. After washed and resuspended by PBS, PKH26-labeled exosomes were added to the well-grown BMECs and incubated together at 37 °C. The fluorescence was observed by confocal microscope 2 and 4 h later after the cells were stained with 1-mg/ml Hoechst (Beyotime) for 15 min and subsequent washing with PBS.

### Cell transfection

Murine miRNA-210-3p mimics, inhibitor and negative control were obtained from GenePharma (Shanghai, China) and transfected into the BMSCs at 70% confluence. The lentivirus was transfected by following the manufacturer’s instructions [50, 51]. In brief, BMSCs were cultured in 6-well plates (1 × 10^5^ cells/well) and incubated with MSC culture medium containing the lentivirus (at 1 × 10^7^ infection-forming units) for 24 h. After that, the medium was refreshed. The efficiency monitored by quantitative reverse transcriptase PCR (qRT-PCR). BMSC-Exos^miRNA−210 mimic^ and BMSC-Exos^miRNA−210 inhibitor^ were obtained using the ultracentrifugation approach from BMSCs after treatment above.

### Administration of exosomes to the GCS-induced ONFH rat model

Eight-week-old female Sprague–Dawley rats (230 ± 20 g) were housed under standard diurnal light/dark conditions, fed with a standard diet and allowed access to tap water ad libitum. Sample size was calculated according to the equation proposed by Mead [52, 53]. Randomization was performed by an independent research assistant using a random number table. The rats were randomly divided into five groups: GCS group (treated with steroids to induce ONFH, n = 7), blank control group (treated with an equal volume of PBS, n = 7), GCS + Exos group (treated with steroids and BMSC-Exos, n = 7), GCS + Exos^miRNA−210 mimic^ group (treated with steroids and BMSC-Exos^miRNA−210 mimic^, n = 7) and GCS + Exos^miRNA−210 inhibitor^ group (treated with steroids and BMSC-Exos^miRNA−210 inhibitor^, n = 7). The ONFH model was created by treatment with steroids as previously described [54, 55]. Briefly, methylprednisolone acetate (MP; Pfizer Manufacturing) (40 mg/kg) was injected intramuscularly for three times per week for 3 weeks to induce ONFH. Before each MP injection, tail vein injection was performed with PBS [100 ul], BMSC-Exos [100 ul; 10^11^ particles/ml], BMSC-Exos^miRNA−210 mimic^ [100 ul; 10^11^ particles/ml] and BMSC-Exos^miRNA−210 inhibitor^ [100 ul; 10^11^ particles/ml]. After completing the course of injections, the rats were fed a standard diet and allowed free activity for another 3 weeks. Then, the femoral heads of all the rats were collected for further study.

### Macroscopic and histologic analyses

Behavioral evaluations were adopted to evaluate joint pain and spontaneous activity levels of rats in different groups as previously described [56–58], and rats in blank control group and GCS + Exos^miRNA−210 mimic^ group showed less pain and higher activity levels. After behavioral evaluations and imaging evaluations by micro-CT, rats were sacrificed, and femoral heads were harvested. Then, femoral heads were rapidly imaged and evaluated by its morphology and surface appearance of cartilage. For histological analysis, the samples were fixed in 4% formaldehyde, decalcified in 10% EDTA solution and then embedded in paraffin. The embedded samples were cut into 5-μm sections with a microtome (Leica Microsystems). Sections were stained with hematoxylin and eosin (HE) and visualized under a light microscope (Leica Microsystems). The extent and severity of osteonecrosis was evaluated according to changes in the trabecular bone by two researchers who were blinded to the identity of the samples. Local osteonecrosis was determined according to the presence of empty lacunae or pyknotic nuclei of osteocytes within the trabecular bone, as well as the thickness and density of the trabecular bone.

### Micro-CT analysis

After dissection of the soft tissue, the femoral heads of the rats were fixed overnight in 70% ethanol at 4 °C and analyzed by micro-CT (Quantum GX II; PerkinElmer micro-CT) under high-resolution mode. Micro-CT scans were acquired over a scan time of 14 min, with a voltage of 80 kV, a current of 100 mA and a pixel size of 90 mm. A horizontal view of the entire subchondral bone of femoral heads was used for three-dimensional histomorphometric analysis, with bone volume/tissue volume (BV/TV; %), trabecular separation (Tb.Sp; um), trabecular thickness (Tb.Th; um) and trabecular number (Tb.N; 1/mm) extracted for performing the comparisons.

### Immunofluorescence assay

BMECs and coronal sections of femoral heads were incubated with CD31 (Abcam, 1:100), CD133 (Abcam, 1:100) and vWF (Abcam, 1:50) antibody overnight at 4 °C. Then, the cells and femoral heads sections were incubated with Cy3-conjugated secondary antibodies (1:100; Boster Biological) for 30 min at room temperature in the dark. Then, 5-μg/mL 4′,6‐diamidino‐2‐phenylindole (DAPI; 1:1000; Beyotime) was used to stain the slips for 30 s, rinsing done by PBS after which a confocal laser scanning microscope was used to analyze the results.

### Statistical analysis

All data are shown as mean ± standard deviation (SD). For multiple-group comparisons, one-way analysis of variance (ANOVA) and Kruskal–Wallis tests were performed. Statistical analyses were performed using SPSS 16.0 software (SPSS, Inc., Chicago, IL, USA). *p* values < 0.05 were considered statistically significant.

## Results

### Identification of BMSCs, BMECs and BMSC-Exos

**Fig. 1:**
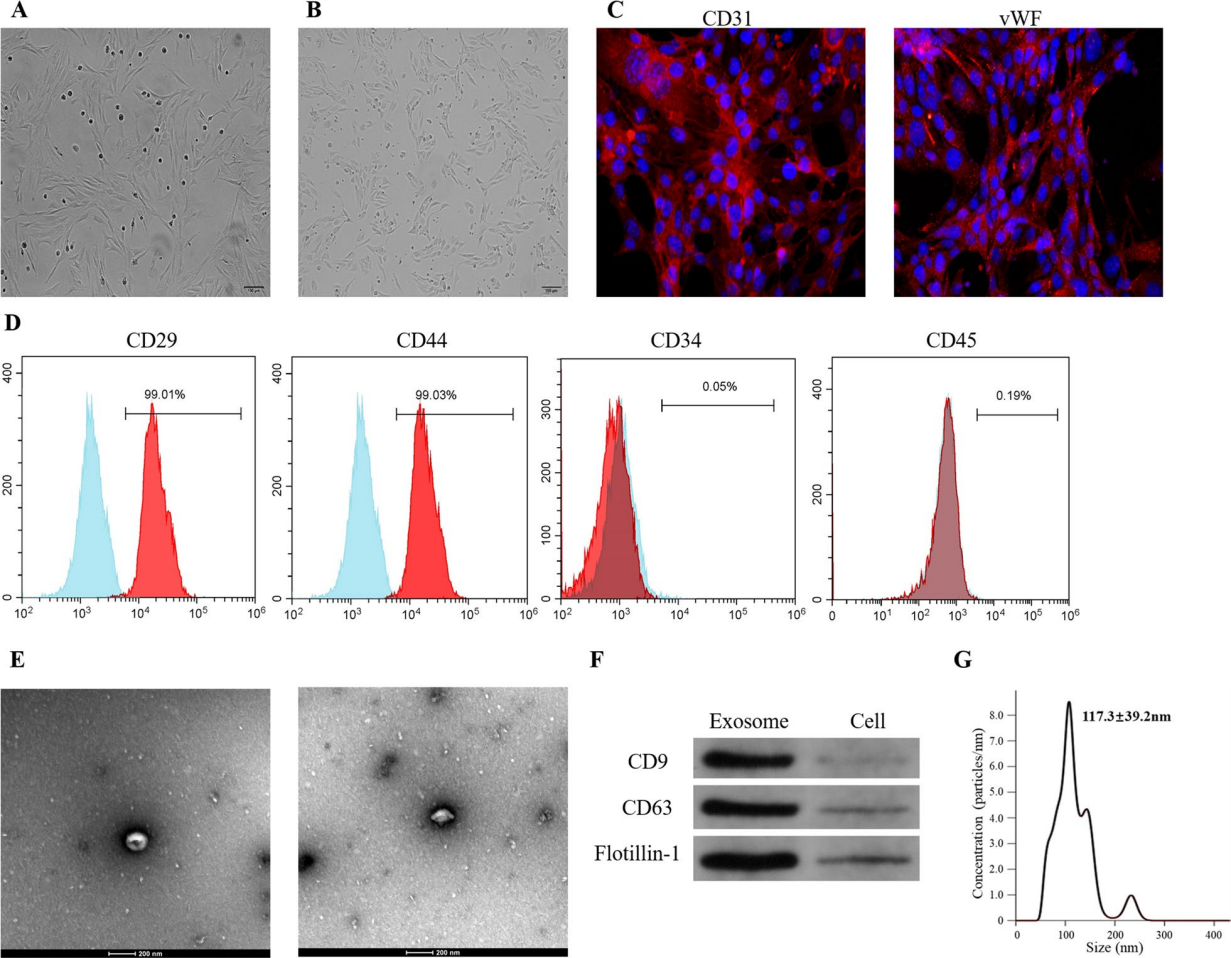
Identification of bone microvascular endothelial cells (BMECs), bone marrow mesenchymal stem cells (BMSCs) and exosomes. **A** Light microscope analyses with the morphology of BMSCs. **B** Light microscope analyses with the morphology of BMECs. **C** Immunofluorescence staining results of CD31 and vWF. **D** Flow cytometry analysis of cell surface markers of BMSCs, including CD29, CD34, CD44 and CD45. **E** Morphology of exosomes under the transmission electron microscopy. **F** Exosome surface markers (CD63, CD9 and flotillin-1) measured by Western blotting. **G** Particle size distribution of the exosomes as measured by a nanoparticle tracking analyzer

Under the microscope, BMSCs showed a uniform, fibroblast-like appearance and had a characteristic spindle shape (Fig. 1A), while BMECs formed adherent colonies and exhibited a cobblestone-like morphology after 10–14 days of culture (Fig. 1B). BMECs were identified by immunofluorescence. The cells highly expressed CD31 and von Willebrand factor (vWF) (Fig. 1C), indicating that these cells were BMECs. Measurement of surface markers on the BMSCs revealed high expression of CD29 and CD44, as well as low expression of CD34 and CD45 (Fig. 1D), which was in line with the criteria for the identification of stem cells [43]. Exosomes derived from BMSCs were successfully isolated, which showed a spherical microvesicle structure under the TEM (Fig. 1E). NTA also demonstrated that the average diameter of the exosomes was 117.3 nm, with a mean concentration of 5.42 × 10^12^ particles/ml (Fig. 1G and Additional file 1: Figure S1). Western blotting analysis detected exosomal surface markers of CD63, CD9 and flotillin-1 (Fig. 1F). These confirmed that the exosomes have been successfully isolated from the BMSCs.

### Protective effect of BMSC-Exos to BMECs in response to GCS treatment

**Fig. 2:**
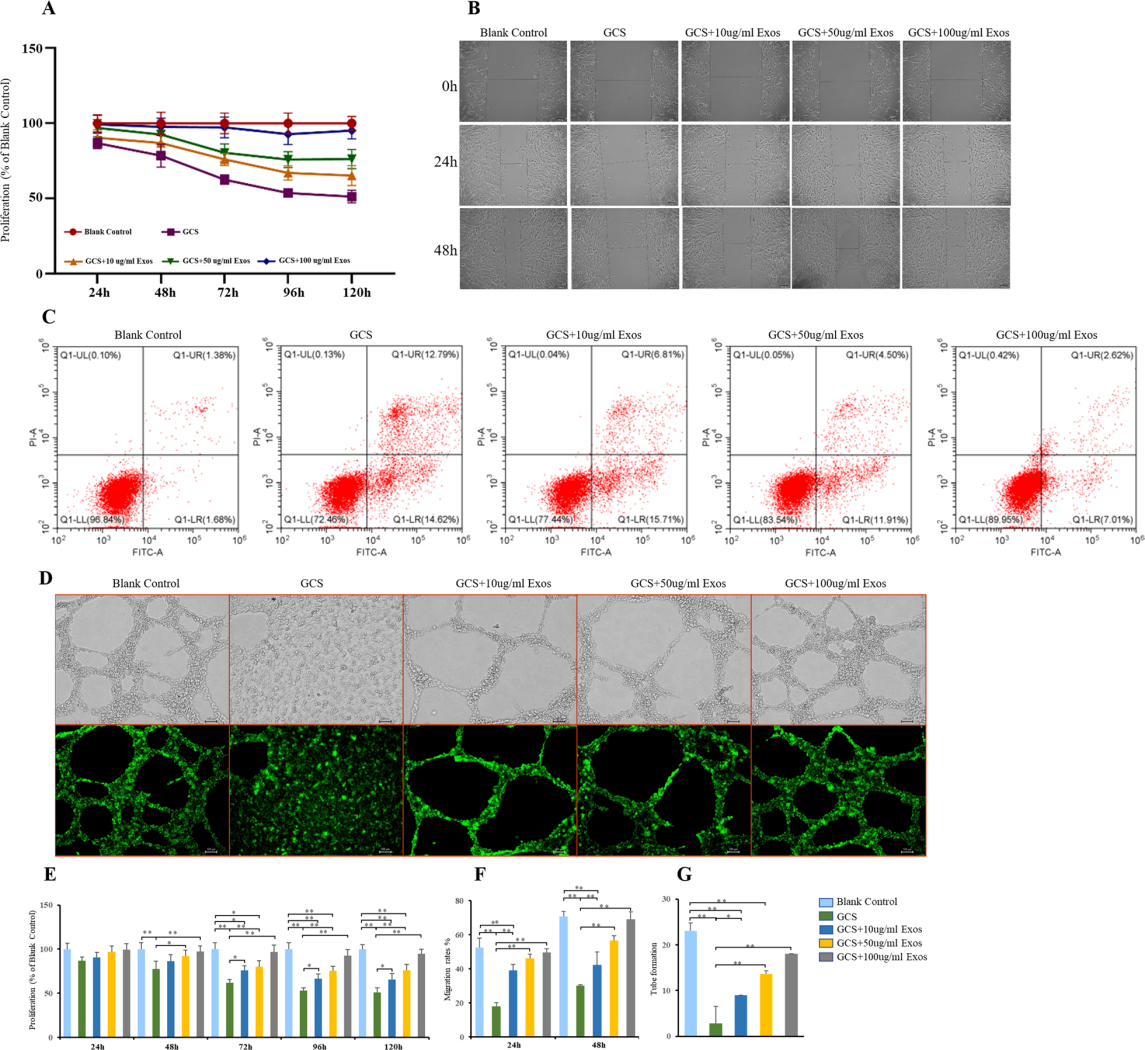
Effects of the exosomes derived from bone marrow mesenchymal stem cells (BMSC-Exos) on BMECs. **A** Cell Counting Kit-8 assays were used to assess the proliferation ability of BMECs. **B** Cell scratch assays were used to assess the migration ability of BMECs. **C** Flow cytometry was used to analyze the apoptosis of BMECs. **D** Tube formation assays were used to assess the angiogenesis ability of BMECs. (E–G) Statistical results; * indicated *p* < 0.05 and ** indicated *p* < 0.01

The 0.1 mg/mL of hydrocortisone was selected to induce glucocorticoid-damaging BMECs in the subsequent experiments as the appropriate concentration. CCK-8 was used to test the cell proliferation activity of BMECs. After the treatment of hydrocortisone, BMECs showed a decreased proliferation activity compared with cells in the blank control group. At 48 h, 72 h, 96 h and 120 h after the treatment of different concentrations of BMSC-Exos, the proliferation activity BMECs increased compared with cell in the GCS treatment group (Fig. 2A and E). Moreover, 100-ug/mL BMSC-Exos could significantly enhance proliferation of the GCS-damaging BMECs (Fig. 2A and E).

Cell scratch assay was used to test the migration ability of BMECs. BMECs after the treatment of GCS showed less migration ability compared with cells in the blank control group at 24 h and 48 h later (Fig. 2B and F). Compared with glucocorticoid-damaging BMECs, BMECs after the treatment of BMSC-Exos showed better migration ability, no matter at 24 h or 48 h later. In addition, high concentration of BMSC-Exos could significantly enhance migration of the GCS-damaging BMECs (Fig. 2B and F).

The Annexin V-FITC/PI kit was used to assess BMECs apoptosis. Flow cytometry demonstrated that the percentage of apoptotic cells increased from 3.16% (blank control group) to 27.54% (GCS group) after treatment with hydrocortisone (Fig. 2C). BMSCs-Exos showed a protective effect on hydrocortisone-induced apoptosis of BMECs. When BMSCs-EVs were added, the percentage of apoptotic cells decreased from 27.54% (GCS group) to 22.56% (GCS + 10 ug/ml Exos group), 16.64% (GCS + 50 ug/ml Exos group) and 10.05% (GCS + 100 ug/ml Exos group) (Fig. 2C).

In the tube formation assay, a smaller tube length and less loop formation were seen in the GCS group. Compared with BMECs in the GCS group, BMECs after the treatment of BMSC-Exos formed more microvessel (Fig. 2D and G). Similarly, BMECs in 100-ug/ml Exos group showed significantly better angiogenesis ability than the GCS-damaging BMECs (Fig. 2D and G). These results indicated that exosomes derived from BMSCs, especially high concentration of BMSC-Exos, can enhance the proliferation, migration, survival and angiogenesis abilities of BMECs.

### BMSC-Exos could be taken in by BMECs and influence the miRNA-210 levels inside

**Fig. 3:**
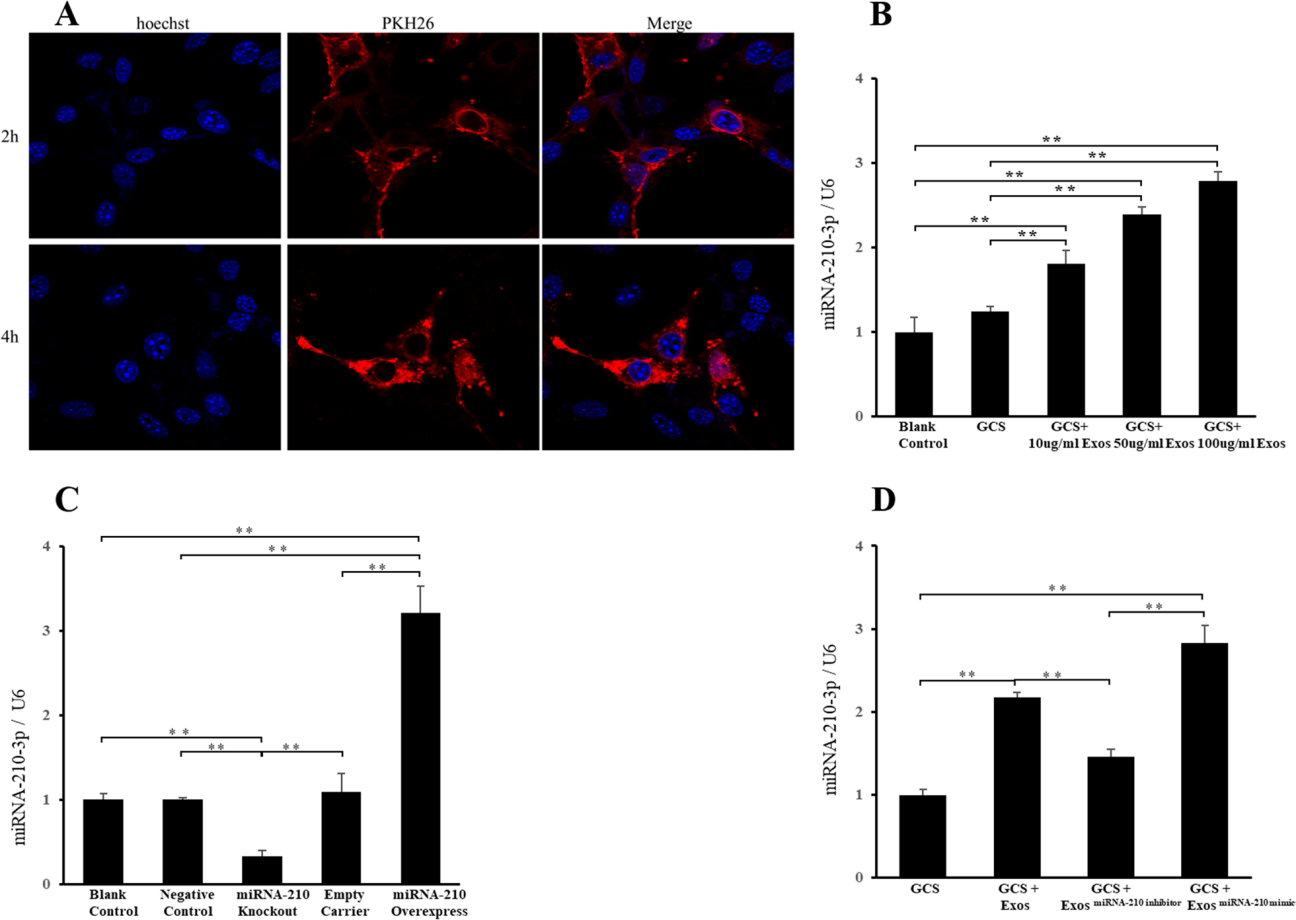
BMSC-Exos could be uptaken by BMECs and influence the level of miRNA-210 inside. **A** Representative immunofluorescence photomicrograph of PKH26–labeled exosomes absorbed by BMECs. **B** miRNA-210 expression in BMECs cocultured with different concentrations of exosomes was analyzed by the quantitative real‐time polymerase chain reaction assay. **C** miRNA-210 expression in each group of BMSCs. **D** miRNA-210 expression in each group of BMECs cocultured with different exosomes

After coculture of BMECs with PKH26-labeled BMSC-Exos for 2–4 h, we observed BMSC-Exos in the cytoplasm of BMECs (Fig. 3A), suggesting that BMSC-Exos were taken by BMECs. To confirm whether BMSC-Exos could deliver miRNA-210 to target BMECs, qRT-PCR was used to detect the expression of miRNA-210 in BMECs after co-incubation. As we expected, miRNA-210 expression in BMSC-Exos treated BMECs was significantly increased compared with BMECs in the GCS and blank control group (Fig. 3B). In addition, the miRNA-210 expression moved up as the concentration of BMSC-Exos increased. miRNA-210 levels were detected by qPCR to ensure successful transfections (Fig. 3C). BMECs in the GCS + BMSC-Exos^miRNA−210 inhibitor^ group showed significantly less expression of miRNA-210 than BMECs in the GCS + BMSC-Exos group, and BMSC-Exos^miRNA−210 mimic^ can significantly improve the miRNA-210 in BMECs (Fig. 3D).

### Importance of miRNA-210 in protective effects of BMSC-Exos to BMECs

**Fig. 4:**
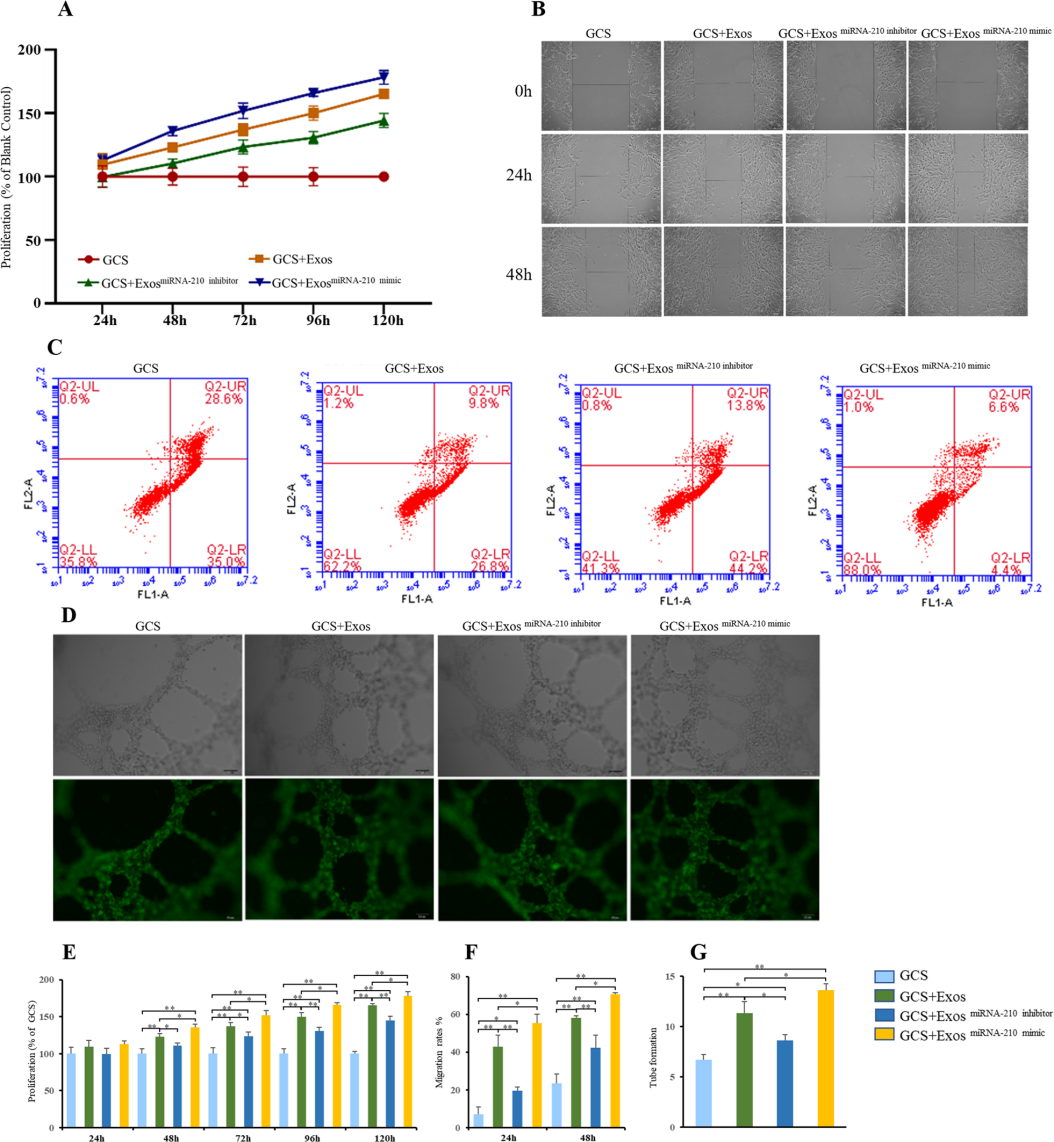
Effects of miRNA-210 in BMSC-Exos on BMECs. Exosomes secreted from bone marrow mesenchymal stem cells transfected with miRNA-210 mimics (BMSC-Exos^miRNA−210 mimic^) or miRNA-210 inhibitor (BMSC-Exos^miRNA−210 inhibitor^) were cocultured with BMECs. **A** Cell Counting Kit-8 assays were used to assess the proliferation ability of BMECs. **B** Cell scratch assays were used to assess the migration ability of BMECs. **C** Flow cytometry was used to analyze the apoptosis of BMECs. **D** Tube formation assays were used to assess the angiogenesis ability of BMECs. **E**–**G** Statistical results; * indicated *p* < 0.05 and ** indicated *p* < 0.01

The effects of the BMSC-Exos, BMSC-Exos^miRNA−210 mimic^ and BMSC-Exos ^miRNA−210 inhibitor^ on the proliferation, migration ability, apoptosis and angiogenesis of GCS-treated BMECs were compared. Compared with BMECs in the GCS + BMSC-Exos group, BMECs in the GCS + BMSC-Exos^miRNA−210 mimic^ group showed significantly higher proliferation activity, while BMECs in the GCS + BMSC-Exos ^miRNA−210 inhibitor^ showed significantly less proliferation activity (Fig. 4A and E). Compared with BMSC-Exos, BMSC-Exos^miRNA−210 mimic^ can significantly improve the migration ability of BMECs (Fig. 4B and F), while BMSC-Exos^miRNA−210 inhibitor^ can reduce the migration ability of BMECs. Similarly, BMECs in the GCS + BMSC-Exos^miRNA−210 mimic^ group showed better angiogenesis ability and survival than BMECs in the GCS + BMSC-Exos group (Fig. 4C, D and G). These results indicated that miRNA-210 in BMSC-Exos played an important role in the protective effects of BMSC-Exos to BMECs, and BMSC-Exos^miRNA−210 mimic^ enhanced the the advantages of BMSC-Exos.

### Effects of BMSC-Exos and miRNA-210 inside in a rat model of GCS-ONFH

**Fig. 5:**
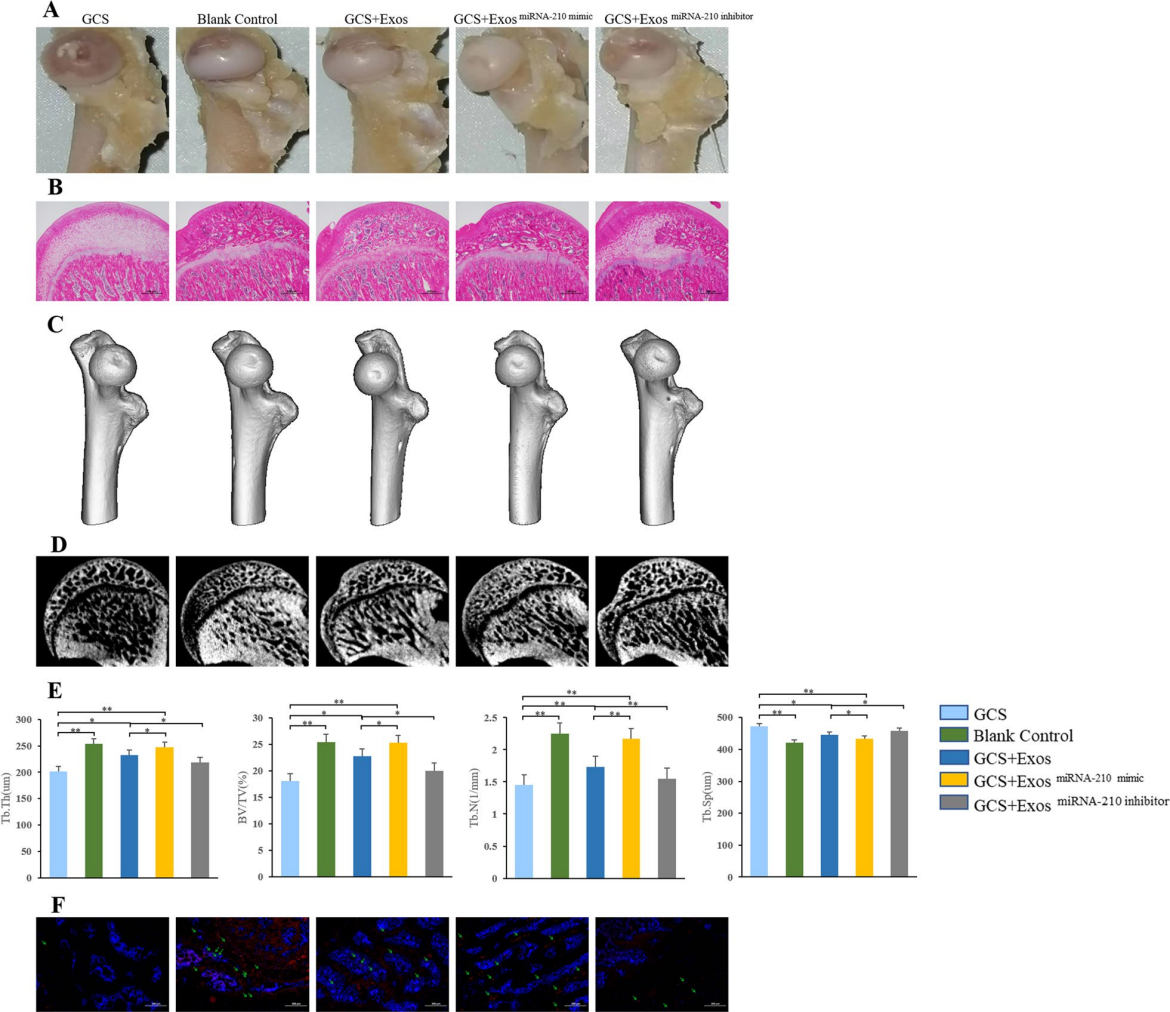
Assessment of trabecular bone structure and angiogenesis in the femoral head of a rat model. **A** Gross observation. **B** Representative sagittal micrographs of hematoxylin and eosin staining. **C** Representative micro-computed tomography (CT) images for the three-dimensional (3D) reconstruction of femoral heads in experimental rats. **D** Representative micro-CT images of femoral heads in the sagittal views. **E** Quantitative micro-CT analysis of femoral heads for trabecular thickness (Tb. Th), bone tissue volume relative to total tissue volume (BV/TV), trabecular number (Tb. N) and trabecular separation (Tb. Sp) (*n* = 5 per group); * indicated *p* < 0.05 and ** indicated *p* < 0.01. **F** Representative immunofluorescence images of CD31-labeled BMECs in the femoral heads to evaluate the angiogenesis of femoral heads

To assess the effect of the BMSC-Exos and miRNA-210 on the progression of GCS-ONFH in vivo, a rat GCS-ONFH model was established. No obvious adverse event was noted. The gross appearance of the femoral heads is shown in Fig. 5A. Collapse of femoral head, surface erosion, pitting or ulceration was observed in the GCS-ONFH rats injected with PBS, indicating that the model was reliable. In the blank control group, the surface cartilage of the femoral head was smooth, the shape of the femoral head was normal, no collapse and no obvious wear were observed. Compared with GCS group, femoral heads of rats in GCS + BMSC-Exos group showed better performance, especially rats after GCS + BMSC-Exos^miRNA−210 mimic^.

Histologic changes in the cartilage were further investigated by H and E staining (Fig. 5B). In the GCS group, HE staining revealed that trabecular bone became sparse and thin. Empty lacunae and pyknosis of osteocytes were observed in the cavities of the trabecular bone. In addition, necrosis of hematopoietic cells and adipose cells was found in the bone marrow. GCS-ONFH rats after the treatment of exosomes showed only slight or no osteonecrosis of the trabecular bone, as well as fewer empty lacunae and adipose cells, especially after BMSC-Exos^miRNA−210 mimic^ treatment.

For micro-CT analysis, a region of interest (ROI) in the femoral head was selected for observation and statistical analysis. In the GCS group, partial collapse of femoral heads could be seen, and the trabecular bone in the femoral head was sparse and relatively thin (Fig. 5C and D). In the GCS + BMSC-Exos group, the trabecular bone showed a better structural integrity (Fig. 5C and D). In quantitative analysis, GCS + BMSC-Exos group showed significantly higher BV/TV, Tb.Th, Tb.N and less Tb.Sp than GCS group. Compared with GCS + BMSC-Exos group, GCS + BMSC-Exos^miRNA−210 mimic^ group showed significantly higher BV/TV, Tb.Th, Tb.N and less Tb.Sp, while GCS + BMSC-Exos^miRNA−210 inhibitor^ group showed less improvement in the structure of trabecular bone (Fig. 5D and E).

Angiogenesis of the femoral head was evaluated via immunofluorescence. The results showed that increased expression CD31 in the femoral head in the GCS + BMSC-Exos group compared with the GCS group (Fig. 5F). Similarly, overexpressing miRNA-210 in BMSC-Exos could enhance the angiogenesis effects of BMSC-Exos, while silencing miRNA-210 in BMSC-Exos resulted in less angiogenesis effects.

## Discussion

In this study, we found that exosomes secreted by normal BMSCs showed strong ability of promoting angiogenesis and repressing the progress of GCS-ONFH, and these effects were all enhanced as the concentration of exosomes increases. Interestingly, exosomes could improve the level of miRNA-210 in BMECs. Considering the important role of miRNA-210 in promoting angiogenesis and the pathogenesis of GCS-ONFH, we overexpressed and silenced miRNA-210 in the exosomes and confirmed that the protective ability to BMECs and GCS-ONFH was enhanced by overexpressing miRNA-210 in BMSC-Exos.

A variety of studies have shown that endothelial cell injury and dysfunction are closely associated with the pathogenesis of ONFH. Feng et al. found that the numbers and functions of circulating endothelial progenitor cells are reduced in GCS-ONFH patients [13]. Similarly, Yu et al. confirmed decreased angiogenic and increased apoptotic activities of bone microvascular endothelial cells in patients with GCS-ONFH [59]. Glucocorticoids can directly injure endothelial cells [60], decrease blood flow to the femoral head [61] and compromise microcirculation [62]. Eventually, this leads to ischemia and hypoxia which cause avascular necrosis of the femoral head and impairs the bone function and structure. Consistent with the previous findings, our in vitro results showed that glucocorticoid inhibited angiogenesis of BMECs, including migration, wound healing and tube formation. Furthermore, we found that BMSC-Exos had a protective role against the negative effects of glucocorticoid on BMECs, which demonstrates that BMSC-Exos have the potential to promote angiogenesis in GCS-ONFH. In this study, we isolated BMECs from the subchondral bone of the femoral head and observed that the isolated cells formed adherent colonies and exhibited a typical cobblestone‐like morphology of endothelial cells. When exposed to extracellular matrix protein, endothelial cells in vitro will form capillary‐like tubules [63], and we found that BMECs could similarly form a capillary tubular pattern when incubated on the Matrigel. In addition, the standard markers of endothelial cells such as CD 31 and vWF were highly expressed as revealed by the immunofluorescence staining. All these confirmed the presence of endothelial cells in the isolates of the subchondral bone of the femoral head. To date, many studies have used human umbilical vein endothelial cells (HUVECs) to replicate the ONFH endothelial condition in vitro [52, 64]. However, HUVECs are derived from a macrovascular bed that does not exist in adults [8]. Therefore, we used BMECs to enable a more specific molecular study instead of using the non‐specific HUVECs in our study, which might better support the results of subsequent animal experiments and molecular mechanism studies.

Stem cell transplantation has been shown to improve the local circulation of ONFH by releasing various cytokines to promote angiogenesis. Tao et al. found that exosomes derived from human platelet-rich plasma prevent apoptosis induced by glucocorticoid-associated endoplasmic reticulum stress in rat osteonecrosis of the femoral head [65]. Zuo et al. found that exosomes derived from human CD34(+) stem cells transfected with miR-26a prevent glucocorticoid-induced osteonecrosis of the femoral head by promoting angiogenesis and osteogenesis [66]. In terms of therapeutic potential of exosomes derived from bone marrow mesenchymal stem cells to GCS-ONFH, there were limited studies. However, BMSC-Exos have to been proved to be effective in ischemic diseases. Arslan et al. found that BMSC-Exos could enhance myocardial viability and prevent adverse remodeling after myocardial ischemia/reperfusion injury [67]. Xin et al. found that systemic administration of BMSC-Exos promote functional recovery and neurovascular plasticity after stroke in rats [68]. In this study, we found that BMSC-Exos could protect BMECs against GCS-induced injuries and confirmed that BMSC-Exos were still effective in promoting angiogenesis in GCS-ONFH.

The most important function of exosomes was their role in communication from host cells to target cells [45]. Exosomes transport a variety of proteins, lipids, RNA and other substances to the site of injury and play an important role in angiogenesis, anti-apoptosis and anti-inflammatory responses [69, 70]. As for the pathogenesis of GCS-ONFH, several studies found that there might be a relationship between miRNA-210 and GCS-ONFH [40, 41, 71]. However, none of them have further studied the relationship and determined the potential therapeutic effects of miRNA-210 to GCS-ONFH. Our results showed that miRNA-210 played an important role in the therapeutic effects of BMSC-Exos to GCS-induced ONFH and BMECs injuries. BMSC-Exos could upregulated the expression of miRNA-210 in BMECs, and overexpressing miRNA-210 in BMSC-Exos could further enhance the proliferation and migration ability of BMECs and inhibit their apoptosis in response to GCS treatment. In vivo results also showed that miRNA-210 in BMSC-Exos was important in improving structure of trabecular bone and number of vascular endothelial cells in femoral heads. Interestingly, Zhang et al. found that targeted delivery of miRNA-210 provides an angiogenic effect to the ischemic brain and could be a treatment for ischemic stroke [39], and Besnier et al. found that miRNA-210 modulated circulating proangiogenic cells and improved their therapeutic potential in limb ischemia, which was similar to our results that miRNA-210 could improve angiogenesis and ameliorate glucocorticoid-induced osteonecrosis of femoral head.

We have to acknowledge that there were several limitations in this work. First, in this study, the effects of 10-ug/ml, 50-ug/ml and 100-ug/ml BMSC-Exos on BMECs were tested, but the optimal concentration of BMSC-Exos to BMECs was not determined. Second, miRNA mainly regulates gene expression by creating a complementary base pair with the 3’-untranslated region (UTR) of the target mRNA [72]. In this study, a dual-luciferase reporter assay was not used to verify the target gene of miRNA-210 in alleviating BMECs injury and the progression of GCS-induced ONFH. Thus, signaling pathways and molecular mechanisms of miRNA-210 need to be further studied. Third, the number of rats in each group is relatively small in the animal experiment, and the animal sample size needs to be expanded.

In conclusion, this study determined the important role of BMECs in the pathogenesis of GCS-induced ONFH and confirmed the therapeutic value of BMSC-Exos. In addition, we found that miRNA-210 in exosomes could serve as a new target for treating GCS-induced ONFH.

### Supplementary Information

**Additional file 1: Table S1.** Primer sequences for qRT-PCR analysis; **Figure S1.** Particle size distribution of exosomes measured by a nanoparticle tracking analyzer; **Figure S2.** Morphology of exosomes under the transmission electron microscopy; **Figure S3.** Exosome surface markers measured by western blotting.

## Data Availability

The data are available from the corresponding author upon request.
