# Supplementary material for: Exosomes from bone marrow mesenchymal stem cells ameliorate glucocorticoid-induced osteonecrosis of femoral head by transferring microRNA-210 into bone microvascular endothelial cells
Source: J Orthop Surg Res. 2023 Dec 7;18:939. doi: 10.1186/s13018-023-04440-x (PMC10704824; doi:10.1186/s13018-023-04440-x)

Supplementary Material

Exosomes from bone marrow mesenchymal stem cells ameliorate glucocorticoid-induced osteonecrosis of femoral head by transferring microRNA-210 into bone microvascular endothelial cells

Table S1. Primer sequences for qRT-PCR analysis.

rno-miR-210-3p-F: ACACTCCAGCTGGGCTGTGCGTGTGACAGC
rno-miR-210-3p-R: CTCAACTGGTGTCGTGGAGTCGGCAATTCAGTTGAGTCAGCCGC
U6-F: CTCGCTTCGGCAGCACA
U6-R: AACGCTTCACGAATTTGCGT

Figure S1. Particle size distribution of the exosomes as measured by a nanoparticle tracking analyzer.

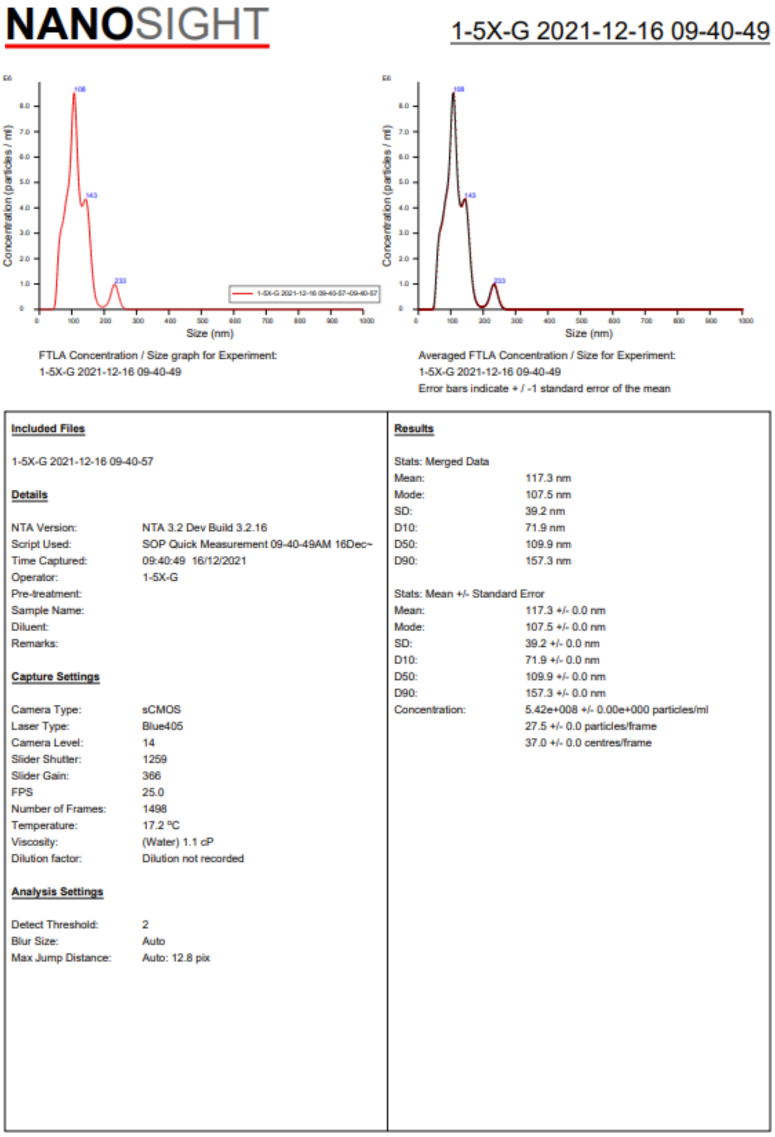

Figure S2. Morphology of exosomes under the transmission electron microscopy

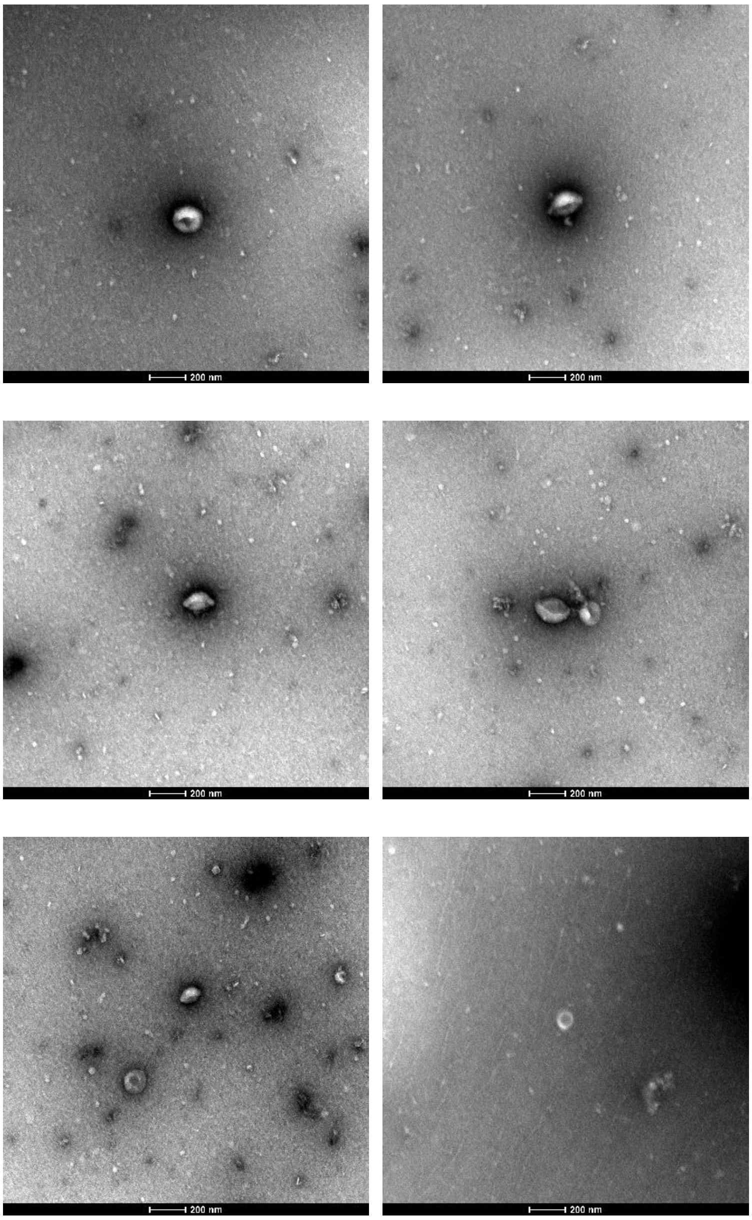

Figure S3. Exosome surface markers measured by Western blotting.

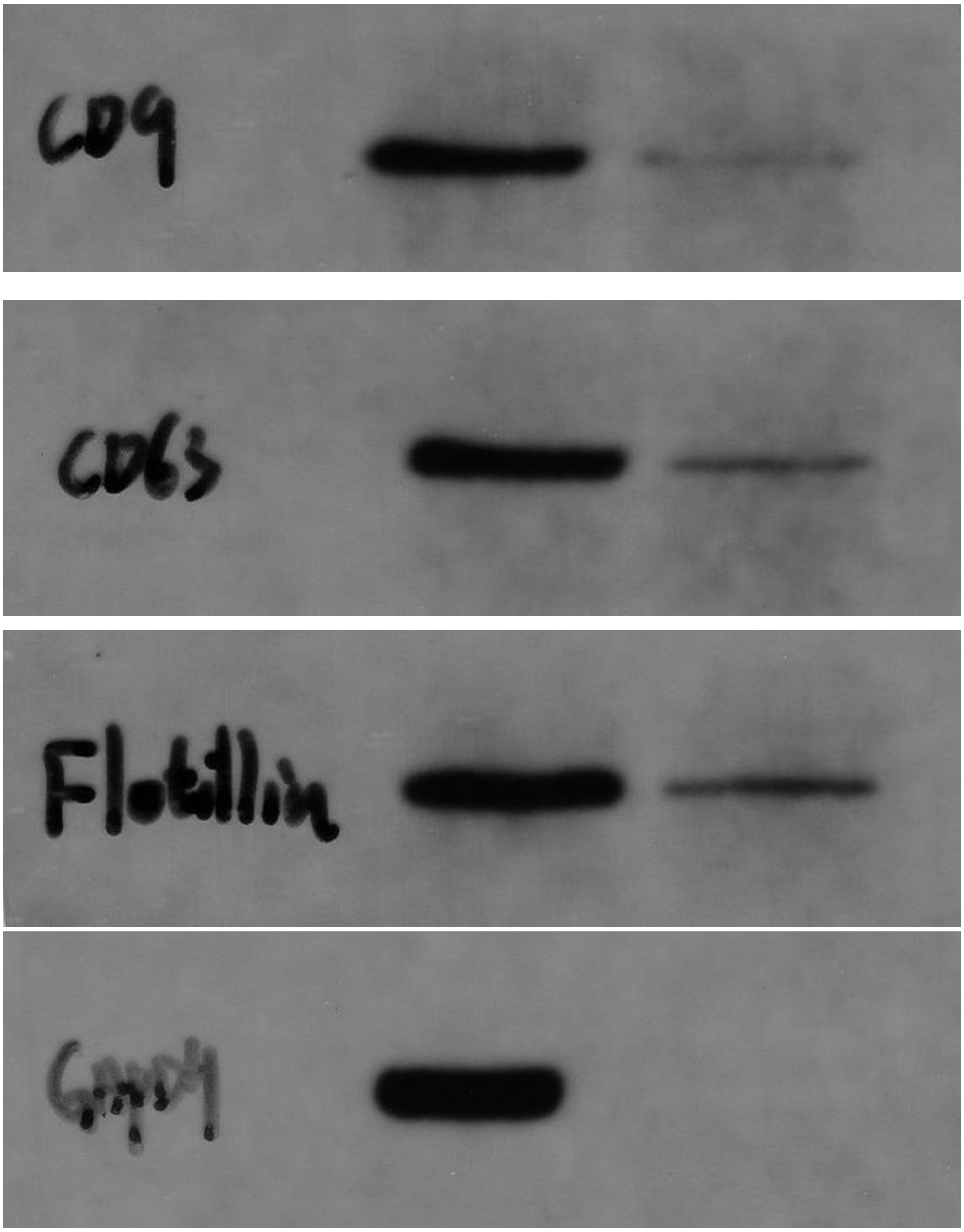

Supplement: Supplementary file 1 — Additional file 1: Table S1. Primer sequences for qRT-PCR analysis; Figure S1. Particle size distribution of exosomes measured by a nanoparticle tracking analyzer; Figure S2. Morphology of exosomes under the transmission electron microscopy; Figure S3. Exosome surface markers measured by western blotting. [file 13018_2023_4440_MOESM1_ESM.pdf]
